# Detailed Balance = Complex Balance + Cycle Balance: A Graph-Theoretic Proof for Reaction Networks and Markov Chains

**DOI:** 10.1007/s11538-020-00792-1

**Published:** 2020-09-03

**Authors:** Stefan Müller, Badal Joshi

**Affiliations:** 1 Faculty of Mathematics, University of Vienna, Vienna, Austria; 2 Department of Mathematics, California State University San Marcos, San Marcos, CA USA

**Keywords:** Chemical reaction network, Arbitrary kinetics, Graph theory, Induced graph, Mixed graph

## Abstract

We further clarify the relation between detailed-balanced and complex-balanced equilibria of reversible chemical reaction networks. Our results hold for arbitrary kinetics and also for boundary equilibria. Detailed balance, complex balance, “formal balance,” and the new notion of “cycle balance” are all defined in terms of the underlying graph. This fact allows elementary graph-theoretic (non-algebraic) proofs of a previous result (detailed balance = complex balance + formal balance), our main result (detailed balance = complex balance + cycle balance), and a corresponding result in the setting of continuous-time Markov chains.

## Introduction

Detailed balance and complex balance are important concepts in chemical reaction network theory (CRNT). Both principles have been proposed already in the 1870s and 1880s by Ludwig Boltzmann in the kinetic theory of gases (where complex balance is called semi-detailed balance) (Boltzmann 1872, 1887). Around 1900, Rudolf Wegscheider introduced the principle of detailed balance in the field of chemical kinetics (and obtained the necessary conditions on the rate constants named after him) (Wegscheider 1901). Only in the 1970s, Horn and Jackson developed the concept of complex balance (as a generalization of detailed balance) in modern CRNT (Horn and Jackson 1972).

Complex-balanced (CB) mass-action systems display remarkably robust dynamics. If one positive equilibrium is CB, then so is every other equilibrium, which justifies calling the entire system CB. Moreover, there is exactly one positive equilibrium in every stoichiometric class (invariant set), and this equilibrium is asymptotically stable (implied by a strict Lyapunov function) (Horn and Jackson 1972). In various important cases, it has been shown that positive CB equilibria are globally stable (Anderson 2011; Craciun et al. 2013), a property that is conjectured to hold for all CB systems (Horn 1974; Craciun 2015). Finally, mass-action systems that are not CB may be dynamically equivalent to CB systems and have all their strong properties (Craciun et al. 2020).

For mass-action kinetics, complex balance has been characterized by Horn (1972), and explicit conditions on the “tree constants” of the underlying graph have been provided by Craciun et al. (2009); see also (Johnston 2014; Müller and Regensburger 2014). Detailed balance has been characterized by Feinberg (1989) and Schuster and Schuster (1989). Feinberg obtains two classes of conditions on the equilibrium constants: $\gamma = r - m + \ell$ “circuit conditions” and $\delta = m - \ell - s$ “spanning forest conditions.” Thereby, $\delta$ is the deficiency of the network Feinberg (1972/73), and $\gamma$ is the cycle rank (cyclomatic number) of the underlying (undirected) graph (Berge 1962). That is, *r* is the number of reversible reactions (pairs of edges), *m* is the number of complexes (vertices), $\ell$ is the number of linkage classes (connected components), and *s* is the rank of the stoichiometric matrix. Schuster and Schuster consider “generalized mass-action kinetics” in the sense that the net reaction rate contains a mass-action factor (as for enzyme kinetics). They provide “generalized Wegscheider’s conditions” on the equilibrium constants; in fact, they obtain $r-s \; (=\gamma +\delta )$ independent conditions. Finally, Dickenstein and Perez-Millan have shown that, given the circuit conditions (“formal balance”), the conditions on the tree constants (complex balance) agree with the spanning forest conditions on the equilibrium constants (detailed balance). That is, detailed balance is equivalent to complex balance plus formal balance, and the result can be extended from mass action to “general kinetics” (Dickenstein and Pérez Millán 2011). For mass action, an alternative proof has been given in van der Schaft et al. (2015). For stochastic mass action, the stationary distribution of the resulting continuous-time Markov chain is a product-form Poisson distribution if and only if the underlying deterministic system is CB (Anderson et al. 2010; Cappelletti and Wiuf 2016). If a CB system is also detailed-balanced, then the stationary solution is detailed-balanced (reversible) (Joshi 2015). For other aspects of detailed and complex balance, see, e.g., (Müller and Hofbauer 2015; Feliu et al. 2018).

In this work, we provide new conditions on a complex-balanced equilibrium of a reversible chemical reaction network to be detailed-balanced. As just stated, a characterization has already been obtained in Dickenstein and Pérez Millán (2011). On the one hand, we give an elementary graph-theoretic (non-algebraic) proof of the previous result (without using the conditions on the tree/equilibrium constants for complex/detailed balance). On the other hand, we show that complex balance plus a condition significantly weaker than formal balance, namely the absence of directed cycles in an induced (mixed) graph, is equivalent to detailed balance. The result immediately holds for arbitrary kinetics and also for boundary equilibria. Since our proof is based on the induced graph, it can be applied in other settings with an underlying graph structure. We illustrate this via continuous-time Markov chains.

The work is organized as follows. First, we present the elementary argument (balance in mixed graphs) that is common to all types of networks. Then, we apply it to different types of networks (balance in reaction networks and balance in Markov chains).

## Balance in Mixed Graphs

The object of the study in this section is a simple mixed graph. Recall that a *mixed* graph contains undirected and directed edges, in general, and that a *simple* mixed graph does not contain multiple edges (connecting two vertices) or loops (connecting a vertex to itself).

Let $G=(V,U,D)$ be a simple mixed graph (with vertices *V*, undirected edges *U*, and directed edges *D*). Explicitly, if two vertices $v, v'\in V$ are connected by an edge, then $v \ne v'$ and exactly one of the following holds: $(v {\; -- \;}v') \in U$, $(v \rightarrow v') \in D$, or $(v \leftarrow v') \in D$.

A *path* is a (finite or infinite) sequence of edges which connect a sequence of distinct vertices. For finite paths, the first and last vertex may be identical, in which case the path is a *cycle*. A path is called *directed* if it contains only directed edges and all edges have the same direction (along the path). In other words, a path connecting the vertices $v,v',v'',\ldots$ is directed if $v \rightarrow v' \rightarrow v'' \rightarrow \ldots$ or $v \leftarrow v' \leftarrow v'' \leftarrow \ldots$. A path is called *weakly directed* if it contains a directed edge and all directed edges have the same direction.

An edge is called *balanced* if it is undirected. A vertex is called *balanced* if the set of incident edges contains either only undirected edges or a pair of oppositely directed edges (with respect to the vertex). In other words, a vertex *v* is balanced if the existence of $v'$ with $v' \rightarrow v$ implies the existence of $v''$ with $v \rightarrow v''$ and vice versa. Note that $v' \ne v''$ by the simplicity of the graph.

*G* is called *edge-balanced/vertex-balanced* if every edge/vertex is balanced.

### Finite Graphs

An edge-balanced graph has only undirected edges and therefore is vertex-balanced and contains no directed cycle. In the following, we show the converse.

#### Proposition 1

Let $G=(V,U,D)$ be a finite, simple mixed graph. If *G* is vertex-balanced, but not edge-balanced, then it contains a directed cycle.

#### Proof

Assume that *G* is vertex-balanced and that there exists a directed edge $v \rightarrow v'$. By vertex balance for $v'$, there exists a corresponding directed edge $v' \rightarrow v''$. Repeating this argument, we construct a directed path $v \rightarrow v' \rightarrow v'' \rightarrow \ldots$ which, by the finiteness of the graph, eventually yields a directed cycle. $\square$

The main result used in the following section is the contrapositive of Proposition 1, which we state as a theorem.

#### Theorem 1

Let $G=(V,U,D)$ be a finite, simple mixed graph. If *G* is vertex-balanced and contains no directed cycle, then it is edge-balanced.

### Infinite Graphs

A directed path is called *bi-infinite* if it connects a bi-infinite sequence of vertices. Bi-infinite directed paths can be viewed as a “directed cycles of infinite length.”

#### Proposition 2

Let $G=(V,U,D)$ be a simple mixed graph. If *G* is vertex-balanced, but not edge-balanced, then it contains a directed cycle or a bi-infinite directed path.

#### Proof

Analogous to the proof of Proposition 1. $\square$

Again, as a main result, we state its contrapositive.

#### Theorem 2

Let $G=(V,U,D)$ be a simple mixed graph. If *G* is vertex-balanced and contains no directed cycle or bi-infinite directed path, then it is edge-balanced.

As a consequence, if *G* is vertex-balanced and contains no directed cycle, then it cannot have a finite number of directed edges.

## Balance in Reaction Networks

In the following, we denote the positive real numbers by $\mathbb {R}_{>}$ and the nonnegative real numbers by $\mathbb {R}_{\ge }$. For a vector $x \in \mathbb {R}^n$, we denote its support by ${{\,\mathrm{supp}\,}}(x) = \{ i \mid x_i \ne 0 \}$. For $x,y \in \mathbb {R}^n_\ge$, we define $x^y = \prod _{i=1}^n (x_i)^{y_i} \in \mathbb {R}_\ge$.

A *chemical reaction network* (*G*, *y*) is given by a finite, simple directed graph $G=(V,\mathcal {R})$ and a map $y :V \rightarrow \mathbb {R}^n_\ge$. To each vertex $i \in V$, a vector *(complex)* ${y(i)} \in \mathbb {R}^n_\ge$ is assigned. Complexes represent formal sums of *n* chemical species which are the left- and right-hand sides of chemical reactions.

As an example, consider the “network” consisting of the single reaction $\mathsf A + B \rightarrow C$, involving the three species $\mathsf A, B, C$. The underlying graph has two vertices, say 1 and 2, and one edge, $1 \rightarrow 2$, that is, $G=(\{1,2\},\{1\rightarrow 2\})$. The left-hand side of the reaction is a formal sum of species $\mathsf A$ and $\mathsf B$, and the right-hand side equals species $\mathsf C$, that is, they are represented by the complexes $y(1)=(1,1,0)^T$ and $y(2)=(0,0,1)^T$, respectively.

A *kinetic system* (*G*, *y*, *r*) is given by a chemical reaction network (*G*, *y*), where $G=(V,\mathcal {R})$, and a map $r :\mathcal {R}\rightarrow (\mathbb {R}^n_\ge \rightarrow \mathbb {R}_\ge )$. To each edge $(i \rightarrow j) \in \mathcal {R}$, a rate function *(kinetics)* $r_{i \rightarrow j} :\mathbb {R}^n_\ge \rightarrow \mathbb {R}_\ge$ is assigned.

The resulting dynamical system for the concentrations $x \in \mathbb {R}^n_{\ge }$ (of *n* chemical species) is defined as

1 $$\begin{aligned} \frac{\text {d} x}{\text {d} t} = \sum _{(i \rightarrow j) \in \mathcal {R}} \big ( y(j)-y(i) \big ) \, r_{i \rightarrow j} (x) . \end{aligned}$$

### Remark

For “general kinetics,” it is often assumed that $r_{i \rightarrow j}(x) > 0$ if and only if ${{\,\mathrm{supp}\,}}(y(i))\subseteq {{\,\mathrm{supp}\,}}(x)$. Then, $x \in \mathbb {R}^n_>$ implies $r(x)\in \mathbb {R}^\mathcal {R}_>$. For mass-action kinetics, the complexes determine not only the reaction vector $y(j)-y(i)$, but also the reaction rate

$$\begin{aligned} r_{i \rightarrow j}(x) = k_{i \rightarrow j} \, x^{y(i)} \quad \text {for } (i \rightarrow j) \in \mathcal {R}. \end{aligned}$$

In the following, we consider *reversible* reaction networks, where the underlying graph *G* is symmetric, that is, $(i \rightarrow j) \in \mathcal {R}$ if and only if $(j \rightarrow i) \in \mathcal {R}$. For simplicity, we often write *ij* for $i \rightarrow j \in \mathcal {R}$.

### Detailed and Complex Balance

An equilibrium $x \in \mathbb {R}^n_\ge$ of the dynamical system (1) is called *detailed-balanced (DB)* if, for every $ij \in \mathcal {R}$,

$$\begin{aligned} r_{ij}(x) = r_{ji}(x) . \end{aligned}$$

That is, for every (reversible) reaction, the forward and backward rates are equal.

An equilibrium $x \in \mathbb {R}^n_\ge$ of the dynamical system (1) is called *complex-balanced (CB)* if, for every $i \in V$,

$$\begin{aligned} \sum _{ij \in \mathcal {R}} r_{ij}(x) = \sum _{ji \in \mathcal {R}} r_{ji}(x) . \end{aligned}$$

That is, for every complex, the sums of incoming and outgoing rates are equal.

Obviously, we have the implication

2 $$\begin{aligned} x \text { is DB} \quad \implies \quad x \text { is CB}. \end{aligned}$$

### Formal Balance and Other Variants of Cycle Balance

A *directed* cycle $C \subseteq \mathcal {R}$ is a sequence of edges which connect a cyclic sequence of distinct vertices (except that the first and last vertex are identical) and which have the same direction (along the cycle). Reversible reactions are directed two cycles (connecting two vertices), and all cycle conditions below hold trivially for directed two cycles.

A state $x \in \mathbb {R}^n_\ge$ (not necessarily an equilibrium) of the dynamical system (1) is called *formally balanced (FB)* if, for every directed cycle $C \subseteq \mathcal {R}$,

$$\begin{aligned} \prod _{ij \in C} r_{ij}(x) = \prod _{ij \in C} r_{ji}(x) , \end{aligned}$$

cf. (Dickenstein and Pérez Millán 2011). Alternatively, such a state could be called algebraically cycle-balanced; see also the discussion in the setting of Markov chains (Cappelletti and Joshi 2018).

#### Remark

Under quite weak assumptions on the kinetics, formal balance is independent of the state: With every vertex $i \in V$ associate a function $f_i(x)$, with every edge $ij \in \mathcal {R}$ a function $k_{ij} \, g_{ij}(x)$, and assume that the reaction rates can be written as $r_{ij}(x) = k_{ij} \, g_{ij}(x) \, f_i(x)$. Now, let $r(x)\in \mathbb {R}^\mathcal {R}_>$. If $g_{ij}(x) = g_{ji}(x)$ for every $ij \in \mathcal {R}$ or, even more generally, if $\prod _{ij \in C} g_{ij}(x) = \prod _{ij \in C} g_{ji}(x)$ for every directed cycle $C \subseteq \mathcal {R}$, then formal balance amounts to

$$\begin{aligned} \prod _{ij \in C} k_{ij} = \prod _{ij \in C} k_{ji} \end{aligned}$$

for every directed cycle $C \subseteq \mathcal {R}$. For mass action, $f_i(x) = x^{y(i)}$ and $g_{ij}(x) = 1$. For “generalized mass action” in the sense of reversible enzyme kinetics (Schuster and Schuster 1989), $f_i(x) = x^{y(i)}$, $g_{ij}(x) = g_{ji}(x)$, and hence, $r_{ij}(x) - r_{ji}(x) = g_{ij}(x)(k_{ij} \, x^{y(i)}-k_{ji} \, x^{y(j)})$. In both cases, formal balance only depends on the rate constants (for $x\in \mathbb {R}^n_>$).

Formal balance is defined by equations for directed cycles. We introduce two other variants of cycle balance which are defined by inequalities and which are weaker than formal balance.

A state $x \in \mathbb {R}^n_\ge$ of the dynamical system (1) is called *strongly cycle-balanced (sCycB)* if, for every directed cycle $C \subseteq \mathcal {R}$, either $r_{ij}(x) = r_{ji}(x)$ for all $ij \in C$ or there exist $ij \in C$ and $i'j' \in C$ with

$$\begin{aligned} r_{ij}(x) < r_{ji}(x) \quad \text {and} \quad r_{i'j'}(x) > r_{j'i'}(x) . \end{aligned}$$

A state $x \in \mathbb {R}^n_\ge$ of the dynamical system (1) is called *cycle-balanced (CycB)* if, for every directed cycle $C \subseteq \mathcal {R}$, there exist (not necessarily distinct) $ij \in C$ and $i'j' \in C$ with

$$\begin{aligned} r_{ij}(x) \le r_{ji}(x) \quad \text {and} \quad r_{i'j'}(x) \ge r_{j'i'}(x) . \end{aligned}$$

For arbitrary kinetics, we have the implications3Thereby, implication $(*)$ holds for $r(x)\in \mathbb {R}^\mathcal {R}_>$. All other implications hold for $r(x)\in \mathbb {R}^\mathcal {R}_\ge$ (possibly involving zero reaction rates), that is, for all $x \in \mathbb {R}^n_\ge$.

The implication “*x* is FB $\Rightarrow$ *x* is CycB” is obvious if $r(x)\in \mathbb {R}^\mathcal {R}_>$. Otherwise, consider a directed cycle $C \subseteq \mathcal {R}$ and $r_{ij}(x)=0$ for some $ij \in C$. Now, “*x* is FB” implies $r_{j'i'}(x)=0$ for some $i'j' \in C$, and hence, $0=r_{ij}(x) \le r_{ji}(x)$ and $r_{i'j'}(x) \ge r_{j'i'}(x)=0$, that is, “*x* is CycB.” All other implications are obvious.

#### Remark

For “general kinetics,” where $r_{ij}(x) > 0$ if and only if ${{\,\mathrm{supp}\,}}(y(i))\subseteq {{\,\mathrm{supp}\,}}(x)$, in particular, for mass-action kinetics, implication $(*)$ in (3) holds for $x \in \mathbb {R}^n_\ge$.

To see this, first note that the sign of $r_{ij}(x)$ is determined by ${{\,\mathrm{supp}\,}}(y(i))$ and hence by vertex *i* only. If ${{\,\mathrm{supp}\,}}(y(i))\subseteq {{\,\mathrm{supp}\,}}(x)$, we write $r_{i*}(x)>0$ (meaning that $r_{ij}(x)>0$ for all *j* with $ij \in \mathcal {R}$); otherwise, we write $r_{i*}(x)=0$.

Obviously, implication $(*)$ in (3) holds for $x \in \mathbb {R}^n_>$. It remains to consider a directed cycle $C \subseteq \mathcal {R}$ with $r_{ij}(x)=0$ for some $ij \in C$. If $r_{i'j'}(x)=0$ for all $i'j' \in C$ (and hence $r_{i'*}(x)=0$ for all vertices $i'$ in *C*), then also $r_{j'i'}(x)=0$ for all $i'j' \in C$, and both “*x* is FB” and “*x* is sCycB.” Otherwise, $r_{i'j'}(x)>0$ for some $i'j' \in C$. In particular, there is a path $j_1 \rightarrow i_1 \rightarrow \ldots \rightarrow i_\ell \rightarrow j_\ell \subseteq C$ involving the complexes $i_l$ with $r_{i_l *} (x)=0$ for $l=1, \ldots , \ell$ and the (not necessarily distinct) complexes $j_1$ and $j_\ell$ with $r_{j_1 *}(x)>0$ and $r_{j_\ell *}(x)>0$. Hence, $0=r_{i_1 j_1}(x)<r_{j_1 i_1}(x)$ and $0 = r_{i_\ell j_\ell }(x) < r_{j_\ell i_\ell }(x)$, and both “*x* is FB” and “*x* is sCycB.”

As stated above, the two new variants of cycle balance are weaker than formal balance, in general. They allow elementary graph-theoretic proofs of a previous result and of a new result which holds for arbitrary kinetics and boundary equilibria; see Theorem 3.

Algorithmically, all variants of cycle balance (including formal balance) are equally costly: the most expensive step is the identification of all cycles in the underlying graph. For mass action (or “generalized mass action” in the sense of reversible enzyme kinetics Schuster and Schuster 1989) and positive states, formal balance only depends on the rate constants. In this case, also (strong) cycle balance does not depend on the state, which may allow to determine the directions of the net reactions; see Example 1.

### The Induced Graph

Given a reversible reaction network, defined by a finite, simple directed graph $G=(V,\mathcal {R})$, and a state $x \in \mathbb {R}^n_\ge$, the *induced graph* $G_x=(V,U,D)$ is a finite, simple mixed graph (with vertices *V*, undirected edges *U*, and directed edges *D*) defined as

$$\begin{aligned} (i {\; -- \;}j) \in U&\quad \text {if } (i \rightarrow j) \in \mathcal {R}\text { and } r_{ij}(x) = r_{ji}(x) ,\\ (i \rightarrow j) \in D&\quad \text {if } (i \rightarrow j) \in \mathcal {R}\text { and } r_{ij}(x) > r_{ji}(x) . \end{aligned}$$

The induced graph contains at most one edge between any two vertices, and hence, cycles in $G_x$ connect three or more vertices.

Let $x \in \mathbb {R}^n_\ge$ be a state of the dynamical system (1) and $G_x$ be the induced graph. From the definitions in Sect. 2, we have the implications

4 $$\begin{aligned} \begin{array}{lrl} x \text { is DB} &{} \iff &{} G_x \text { is edge-balanced,} \\ x \text { is CB} &{} \implies &{} G_x \text { is vertex-balanced,} \\ x \text { is sCycB} &{} \iff &{} G_x \text { does not contain a weakly directed cycle,} \\ x \text { is CycB} &{} \iff &{} G_x \text { does not contain a directed cycle.} \end{array} \end{aligned}$$

Note that the second implication is not an equivalence; see Remark 1.

### Main Results

As stated in the introduction, it was shown in Dickenstein and Pérez Millán (2011) that detailed balance is equivalent to complex balance plus formal balance. We prove that detailed balance is equivalent to complex balance plus cycle balance.

#### Proposition 3

Let $x \in \mathbb {R}^n_\ge$ be an equilibrium of the dynamical system (1). If *x* is CB and CycB, then it is DB.

#### Proof

By the implications (4) and Theorem 1: 
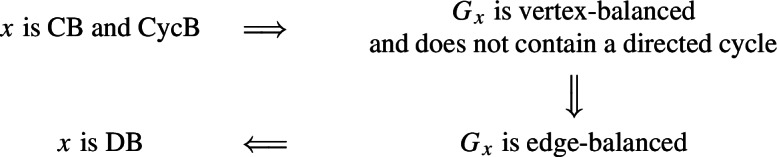
$$\square $$

The above result is new and stronger than the existing result: first, it holds for $x \in \mathbb {R}^n_\ge$; and second, formal balance is stronger than cycle balance, see (3). However, the main advantage from our perspective is its elementary proof, which is entirely graph-theoretic and does not involve any algebraic argument; in particular, it does not assume mass-action kinetics.

To summarize, given complex balance, detailed balance is equivalent to all variants of cycle balance. The result holds for $x \in \mathbb {R}^n_\ge$, that is, also for boundary equilibria.

#### Theorem 3

Let $x \in \mathbb {R}^n_\ge$ be a complex-balanced (CB) equilibrium of the dynamical system (1). The following statements are equivalent:

- *x* is detailed-balanced (DB).
- *x* is formally balanced (FB).
- *x* is strongly cycle-balanced (sCycB).
- *x* is cycle-balanced (CycB).

#### Proof

By the implications (3) and Proposition 3. $\square$

#### Remark 1

Only the second implication in (4) is not an equivalence. In order to obtain an equivalence, we define $x \in \mathbb {R}^n_\ge$ to be *weakly complex-balanced (wCB)* if $G_x$ is vertex-balanced. Then, “*x* is wCB $\Leftrightarrow$ $G_x$ is vertex-balanced,” and Proposition 3 and Theorem 3 also hold if the CB equilibrium is replaced by a wCB equilibrium.

#### Example 1

Consider the reversible cyclic network $G^\triangleright :\mathsf A \rightleftarrows B \rightleftarrows C \rightleftarrows A$ and assume that the (isolated) network follows the laws of thermodynamics. Adding the exchange reactions $\mathsf A \rightleftarrows 0 \rightleftarrows C$ (putting $G^\triangleright$ in a flow reactor) yields the network *G*, which contains two independent cycles; see the left diagram. 
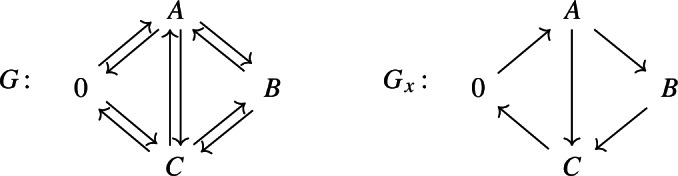


Both networks, $G^\triangleright$ and *G*, have deficiency zero: $\delta ^\triangleright = 3-1-2=0$ and $\delta =4-1-3=0$, respectively. For simplicity, assume mass-action kinetics.

For the isolated network $G^\triangleright$, there exists a complex-balanced equilibrium $x^\triangleright \in \mathbb {R}^3_>$ (implied by $\delta ^\triangleright =0$) which is detailed-balanced (implied by thermodynamics) and hence formally balanced. For any $x\in \mathbb {R}^3_>$, the condition for formal balance is given by $k_\mathsf{A\rightarrow B} \, k_\mathsf{B\rightarrow C} \, k_\mathsf{C\rightarrow A}=k_\mathsf{A\rightarrow C} \, k_\mathsf{C\rightarrow B} \, k_\mathsf{B\rightarrow A}$. Hence, any state $x\in \mathbb {R}^3_>$ is formally balanced and, by (3), (strongly) cycle-balanced. That is, any mixed graph $G^\triangleright _x$, induced by $G^\triangleright$ and *x*, does not contain a (weakly) directed cycle, and the same holds when $G^\triangleright$ is seen as a subnetwork of *G*; see below.

For the full network *G*, there exists a complex-balanced equilibrium $x \in \mathbb {R}^3_>$ (implied by $\delta =0$). Assume that *x* is not detailed-balanced, in particular, that the mixed graph $G_x$, induced by *G* and *x*, does not have $\mathsf C {\; -- \;}0 {\; -- \;}A$ as a subgraph. By complex balance (for the complex $\mathsf 0$), $G_x$ has $\mathsf C \rightarrow 0 \rightarrow A$ (or, alternatively, $\mathsf A \rightarrow 0 \rightarrow C$) as a subgraph; see the right diagram. By Theorem 3, *x* is not cycle-balanced, that is, there exists a directed cycle in $G_x$. By the argument above, the subgraph $G^\triangleright _x$ is **not** a (weakly) directed cycle. 
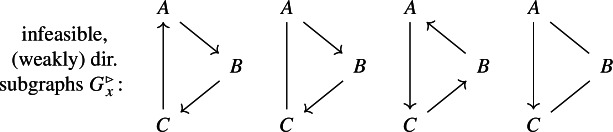
 The only feasible subgraph $G^\triangleright _x$ is $\mathsf C \leftarrow \mathsf A \rightarrow B \rightarrow C$; see again the right diagram above. The induced graph $G_x$ contains the directed cycles $\mathsf 0 \rightarrow A \rightarrow C \rightarrow 0$ and $\mathsf 0 \rightarrow A \rightarrow B \rightarrow C \rightarrow 0$ which involve the exchange reactions (in agreement with thermodynamics).

Remarkably, all edges of the induced graph (all directions of the net reactions) can be determined without computing the complex-balanced equilibrium.

## Balance in Markov Chains

The argument in Sect. 2 has been developed for the application to reaction networks (RNs). However, owing to the abstractness of the result, it is easily applicable in any setting with an underlying graph structure. We illustrate this via Markov chains (MCs), a widely used class of stochastic models with a naturally associated graph.

A continuous-time MC is a random process on a countable state space, where a measure (in particular, a distribution) on the set of states is determined by the initial measure and the transition rates (via the Kolmogorov forward equations). For a formal definition, see, e.g., Norris (1998). In a natural way, states can be viewed as vertices of a directed graph whose edges represent transitions with positive rates.

We denote the set of states (vertices) by *V* and the transition rate from state $x \in V$ to state $y \in V$ by *q*(*x*, *y*). Further, we introduce the set of transitions (edges) $\mathcal {T}$, that is, $(x , y) \in \mathcal {T}$ if $q(x,y) >0$. In the following, we require that $q(x,y) >0$ implies $q(y,x)>0$ for all $x,y \in V$. That is, we consider MCs where the associated simple, directed graph $G=(V,\mathcal {T})$ is symmetric. Such MCs are analogous to reversible RNs, however, we do not refer to them as “reversible” since this term is reserved for another notion; see below.

A measure $\mu$ on the countable set *V* assigns a nonnegative real or infinity to each subset of *V*. Here, we consider only $\sigma$-finite measures where $\mu (\{x\}) < +\infty$ for all $x \in V$. Following standard convention, we drop the curly brackets and write $\mu (x)$ for $\mu (\{x\})$. If $\sum _{x \in V} \mu (x) = 1$, then $\mu$ is a distribution. A measure $\mu$ is *stationary* if, for all $x \in V$,

$$\begin{aligned} \sum _{(x,y) \in \mathcal {T}} \mu (x) q(x,y) = \sum _{(y,x) \in \mathcal {T}} \mu (y) q(y,x) . \end{aligned}$$

A stationary measure of a MC is analogous to a complex-balanced equilibrium of an RN in the sense that, for every state, the sums of incoming and outgoing “probability flows” are equal. Finally, a measure $\mu$ is *reversible* (detailed-balanced) if, for all $(x,y) \in \mathcal {T}$,

$$\begin{aligned} \mu (x) q(x,y) = \mu (y) q(y,x) . \end{aligned}$$

Clearly, the notions of detailed balance in RNs and MCs are analogous.

Given a MC with associated symmetric, simple, directed graph $G=(V, \mathcal {T})$ and a measure $\mu$, the *induced graph* $G_\mu =(V,U,D)$ is a simple, mixed graph defined as

$$\begin{aligned} (x {\; -- \;}y) \in U&\quad \text {if } (x,y) \in \mathcal {T}\text { and } \mu (x) q(x,y) = \mu (y) q(y,x) ,\\ (x \rightarrow y) \in D&\quad \text {if } (x,y) \in \mathcal {T}\text { and } \mu (x) q(x,y) > \mu (y) q(y,x) . \end{aligned}$$

Now, let $\mu$ be a measure of a MC and $G_\mu$ be the induced graph. From the definitions in Sect. 2, we have the implications

$$\begin{aligned} \begin{array}{lrl} \mu \text { is reversible} &{} \iff &{} G_\mu \text { is edge-balanced,} \\ \mu \text { is stationary} &{} \implies &{} G_\mu \text { is vertex-balanced.} \end{array} \end{aligned}$$

An application of Theorem 2 immediately yields the following result.

### Theorem 4

Let *G* be the graph associated with a continuous-time Markov chain, where $q(x,y) >0$ if and only if $q(y,x)>0$. Let $\mu$ be a stationary measure. If the induced graph $G_\mu$ does not contain a directed cycle or a bi-infinite directed path, then $\mu$ is a reversible measure.

Its contrapositive is useful to state. If a stationary measure is not reversible, then the induced graph contains a directed cycle or a bi-infinite directed path. See Examples 2 and 3.

### Example 2

Consider again the reversible cyclic network $\mathsf A \rightleftarrows B \rightleftarrows C \rightleftarrows A$, but this time with **stochastic** mass-action kinetics. The corresponding rate constants are specified as edge labels in the graph below. 
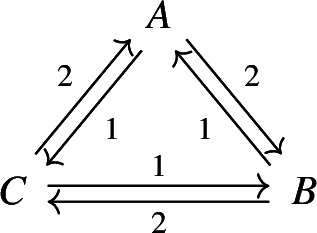
 The (infinite) graph $G=(V,\mathcal {T})$ associated with the Markov chain is given by $V= \mathbb {Z}^3_\ge$, $q\left( (a,b,c)\rightarrow (a-1,b+1,c)\right) =2a$, $q\left( (a,b,c)\rightarrow (a+1,b-1,c)\right) =b$, $q\left( (a,b,c)\rightarrow (a-1,b,c+1)\right) =a$, etc.

For the deterministic system, $x = (1,1,1)$ is a complex-balanced, but not detailed-balanced equilibrium. For the stochastic system, the stationary (necessarily “complex-balanced”) distribution $\pi :\mathbb {Z}^3_\ge \rightarrow \mathbb {R}$ is given by the product form

$$\begin{aligned} \pi (a,b,c) = \frac{\mathrm{e}^{-3}}{a! \, b! \, c!} \, , \end{aligned}$$

cf. Anderson et al. (2010). Since this stationary distribution is not reversible (detailed-balanced), the induced graph $G_\pi$ must have a directed cycle or a bi-infinite directed path. Indeed, the (infinite) induced graph can be decomposed into directed cycles (connecting three vertices), as shown in the graph below. The corresponding net probability flows between states are specified as edge labels. 
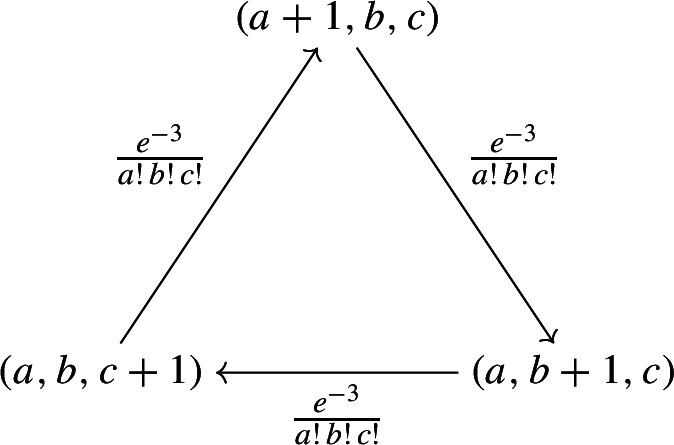


### Example 3

Let $q \in (0,1)$. Consider a Markov chain given by $V = \mathbb {Z}$, $q(x,x+1) = 2q^{-|x|}$ and $q(x,x-1) = q^{-|x|}$ for $x \in \mathbb {Z}$, and $q(x,x')=0$ otherwise. Obviously, there are no directed cycles in the associated graph *G*, except for the trivial two cycles. A stationary distribution on $\mathbb {Z}$ is

$$\begin{aligned} \pi (x) = \pi (0) q^{|x|} \end{aligned}$$

with normalization constant $\pi (0)>0$. However, this distribution is not reversible (detailed-balanced), since $\pi (x)q(x,x+1) \ne \pi (x+1)q(x+1,x)$ for any $x \in \mathbb {Z}$. Hence, the induced graph $G_\pi$ has directed edges $x \rightarrow x+1$ for $x \in \mathbb {Z}$. The induced graph is vertex-balanced, but not edge-balanced, in particular, $G_\pi$ contains a bi-infinite directed path.

Since $q(x,y) > 0$ if and only if $q(y,x)>0$ and there are no (non-trivial) cycles, there must be a reversible stationary measure on $\mathbb {Z}$ as well. In fact,

$$\begin{aligned} \rho (x) = \rho (0) {\left\{ \begin{array}{ll} (2q)^x &{} \text {if } x \ge 0 \\ \left( \frac{q}{2}\right) ^{-x} &{} \text {if } x < 0 \end{array}\right. } \end{aligned}$$

is such a measure. For $q<\frac{1}{2}$, it is finite and hence a distribution (for some normalization constant $\rho (0)>0$). The induced graph $G_\rho$ is both vertex-balanced and edge-balanced.

Since there exist two different stationary distributions $\pi \ne \rho$, the Markov chain is not positive recurrent.

Finally, we summarize similarities and dissimilarities in the settings of RNs and MCs in a table.

**Table:** 

	Chemical **reaction network **with mass-action kinetics	Continuous-time **Markov chain**
Variable	Species concentrations *x*	Probability measure $\mu$
Function on vertex *i*	Monomial $x^{y(i)}$	$\mu (i)$
Function on edge *ij*	Rate constant $k_{ij}$	Transition rate *q*(*i*, *j*)
Product function on edge *ij*	Reaction rate $k_{ij} \, x^{y(i)}$	Probability flow $\mu (i) \, q(i,j)$
Vertex balance	Complex-balanced equilibrium	Stationary measure
Edge balance	Detailed-balanced equilibrium	Reversible measure
Cycle conditions	Formal balance, cycle balance	Kolmogorov cycle conditions
